# Reduction of Behavioral Psychological Symptoms of Dementia by Multimodal Comprehensive Care for Vulnerable Geriatric Patients in an Acute Care Hospital: A Case Series

**DOI:** 10.1155/2016/4813196

**Published:** 2016-03-16

**Authors:** Miwako Honda, Mio Ito, Shogo Ishikawa, Yoichi Takebayashi, Lawrence Tierney

**Affiliations:** ^1^National Hospital Organization Tokyo Medical Center, Meguro, Tokyo 152-0021, Japan; ^2^Tokyo Metropolitan Institute of Gerontology, Itabashi, Tokyo 173-0015, Japan; ^3^Department of Information, Shizuoka University, Hamamatsu, Shizuoka 432-8011, Japan; ^4^University of California San Francisco, San Francisco, CA 94121, USA

## Abstract

Management of Behavioral and Psychological Symptoms of Dementia (BPSD) is a key challenge in geriatric dementia care. A multimodal comprehensive care methodology, Humanitude, with eye contact, verbal communication, and touch as its elements, was provided to three geriatric dementia patients for whom conventional nursing care failed in an acute care hospital. Each episode was evaluated by video analysis. All patients had advanced dementia with BPSD. Failure of care was identified by patient's shouting, screaming, or abrupt movements of limbs. In this case series, conventional care failed for all three patients. Each element of care communication was much shorter than in Humanitude care, which was accepted by the patients. The average of the elements performed during the care was eye contact 0.6%, verbal communication 15.7%, and touch 0.1% in conventional care and 12.5%, 54.8%, and 44.5% in Humanitude care, respectively. The duration of aggressive behavior of each patient during care was 25.0%, 25.4%, and 66.3% in conventional care and 0%, 0%, and 0.3% in Humanitude, respectively. In our case series, conventional care was provided by less eye contact, verbal communication, and touch. The multimodal comprehensive care approach, Humanitude, decreased BPSD and showed success by patients' acceptance of care.

## 1. Introduction

Dementia is a progressive, incurable illness. Among patients with advanced dementia, the final year of life is characterized by a trajectory of persistently severe disability [1]. People with dementia are frequently admitted to the acute care hospital [2], and they are hospitalized two to three times more often than people of the same age without dementia [3]. The management of Behavioral and Psychological Symptoms of Dementia (BPSD) is a key challenge in inpatient dementia care [4], and typical difficulties include the refusal of care. It results in poor quality of care delivered to patients and has raised serious barriers to providing palliative care in geriatrics.

The multimodal comprehensive care—*Humanitude*—is a French-origin methodology for vulnerable elderlies focusing on their perception, emotion, and oral communication [5, 6]. In a prior study, reduction of BPSD by this methodology in a long term care facility was reported [7]. However, the effect in the acute care hospital is not known. We experienced a reduction of BPSD by this multimodal comprehensive methodology for daily care compared to conventional care in the acute care hospital.

Four out of 40 nurses in a medical ward were trained by this methodology. The training consisted of 5-day lecture, followed by bedside teaching by certified instructor as needed basis. The education was supported by the health labour sciences research grant of Japanese Ministry of Health Labour and Welfare. The remaining nurses continued conventional care. When conventional care failed to be delivered, the nurse was replaced and a trained nurse performed the care based on the methodology. Both types of care were recorded and analyzed with the communication modalities: eye contact, verbal communication, and touch. When there was shouting, screaming, or abrupt movement of limbs, it was considered aggressive behavior. Also agitation, refusal of care, disinhibition, or irritability was evaluated by 4 experienced healthcare professionals. Institutional ethics committee approved the video record of care, and written consents were obtained by the patients or family. Videos were analyzed by researchers of informatics based on the standardized scoring system [8].

In this case series, this multimodal care methodology was applied to three advanced Alzheimer's disease patients who had refused care in an acute care hospital.

## 2. Case 1

An 83-year-old woman with Alzheimer's disease was brought to the hospital after an overdose of medication. She had mistaken benzodiazepines for candies and taken a one-month supply of the medication. On admission, she was on memantine 20 mg once a day and quetiapine 25 mg twice a day. Her Mini-Mental State Examination (MMSE) was 3. She was admitted for mental status change and vomiting. In the ward, she refused the care especially taking a shower. The patient was agitated and aggressive to nurses with conventional care. When she was provided a shower, it took 300.5 seconds and the patient spent 25% of care time manifesting aggressive behavior. When multimodal comprehensive care—Humanitude—was applied, the duration was 315.6 seconds and no aggressive behavior was observed all through the care episode. In the comparison of percentage of the modalities during care, eye contact was 1.7% in the conventional care and 19.5% in Humanitude, verbal communication was 19.7% and 73.9%, and touch was 0.2% and 60.4%, respectively (Table 1). Because of the improvement of her symptom, the dosage of quetiapine was reduced to 25 mg once a day.

## 3. Case 2

A 93-year-old woman with Alzheimer's disease had complained of abdominal pain and was admitted to acute care hospital. Cutaneous abscesses of the abdominal wall were found and intravenous antibiotics were started. Her Activity of Daily Living was totally dependent with advanced dementia, and MMSE was not able to be obtained due to inability to communicate the questions. On admission the patient was on sulpiride 50 mg once a day and risperidone as needed basis. In the ward, she continued to scream and act violently. Nurses had difficulty providing care. She refused to change diapers and became increasingly aggressive to the nurses.

During diaper change, conventional care took 360.7 seconds and the patient spent 24.5% of care time evincing aggressive behavior. When Humanitude care was applied, it took 127.6 seconds to complete the care. There was no aggressive behavior and the patient accepted the care peacefully. In the comparison of percentage of the modalities during care, eye contact was 0% in conventional care and 4.3% in Humanitude care, verbal communication was 27.4% and 42.1%, and touch was 0% and 44.0%, respectively (Table 2). After the implementation of the care methodology, risperidone was no more needed.

## 4. Case 3

An 89-year-old woman with Alzheimer's disease was admitted to the acute care hospital because of a urinary tract infection with bacteremia. Her MMSE was 11. Nurses had difficulty providing care. When they attempted to change the diaper with conventional care, she refused aggressively and the delivery of care failed. The attempt ended in 20.5 seconds and 66.3% of the time aggressive behavior was noted. The nurse gave up continuing the care.

When multimodal comprehensive care—Humanitude—was applied, the duration was 241.8 seconds and little (0.26%) aggressive behavior was observed. In the comparison of the modalities, eye contact was 0% in the conventional care and 13.7% in Humanitude, verbal communication was 0% and 48.5%, and touch was 0% and 29.2%, respectively (Table 3).

## 5. Discussion

Three patients with advanced Alzheimer's dementia were difficult to care for in an acute care hospital. BPSD in people with dementia in acute care hospitals is a critical issue in the era of aging society, and the training of medical professionals for adequate skill and technique is crucial.

The multimodal comprehensive care methodology—Humanitude—focuses on perception, emotion, and oral communication. It emphasizes eye contact, verbal communication, and gentle and secure touch during interaction with patients. Our experience with the methodology showed improved acceptance of care. The video analysis revealed that the duration of three modalities of the care was much longer in Humanitude care than in conventional care. The average ratio of eye contact, verbal communication, and touch during the care was 0.6%, 15.7%, and 0.1% in conventional care and 12.5%, 54.8%, and 44.5% in Humanitude care, respectively (Table 4). The duration of conventional care was exceedingly short in Case 3, almost the same in Case 1, and longer in Case 2 comparing to the comprehensive care. In Case 3, the nurse could not continue the care because of strong refusal by the patient and gave up immediately. In Case 2, the care attempt was persisted unsuccessfully and consumed time. Average time of aggressive behavior was 38.6% in conventional care, while it was 0.1% in Humanitude care.

Three modalities are encouraged to be used comprehensively. The subanalysis of the care was done to evaluate simultaneous application of the modalities (Table 5). The conventional care which failed to be accepted by the patients with BPSD had tendency to use no or single modality for care, while Humanitude care employed multiple modalities. It is considered that a multimodal and comprehensive care approach brings successful care to patients with BPSD. It is simple, basic, and effective for patients with dementia to be provided the necessary care during hospital stay.

The importance of delivering adequate palliative care for geriatric patients is widely recognized [9, 10]. For the dementia patients, the acceptance of the care is the key. We believe that a multimodal comprehensive care methodology is effective to deliver care for dementia patients with BPSD.

Other important challenges in geriatrics are polypharmacy and cost effectiveness. After the implementation of Humanitude to a geriatric hospital in France, there was 88.5% reduction of neuroleptics use in 3 years [11]. Results of our case series can generate hypotheses that are useful in designing further studies. The acute care hospital, where this study was conducted, started education program for nurses and outcome measurement research is in progress. Furthermore, with the grant of Japanese Ministry of Health Labour and Welfare, evaluation of this care methodology focusing on family-caregivers as well as long term care facilities is ongoing.

## Figures and Tables

**Table 1:** Time used for the elements of care communication, Case 1.

	Conventional care	Humanitude care
	Second (%)	Second (%)
Total care time	300.5 (100%)	320.2 (100%)
Eye contact	5.3 (1.7%)	62.3 (19.5%)
Verbal communication	59.3 (19.7%)	236.7 (73.9%)
Touch	6.7 (0.2%)	193.3 (60.4%)
Aggressive behavior	75 (25.0%)	0 (0%)
Acceptance of care	No	Yes

**Table 2:** Time used for the elements of care communication, Case 2.

	Conventional care	Humanitude care
	Second (%)	Second (%)
Total care time	360.7 (100%)	127.6 (100%)
Eye contact	0 (0%)	5.5 (4.3%)
Verbal communication	98.8 (27.4%)	53.7 (42.1%)
Touch	0 (0%)	56.1 (44.0%)
Aggressive behavior	88.4 (24.5%)	0 (0%)
Acceptance of care	No	Yes

**Table 3:** Time used for the elements of care communication, Case 3.

	Conventional care	Humanitude care
	Second (%)	Second (%)
Total care time	20.5 (100%)	241.8 (100%)
Eye contact	0 (0%)	33.1 (13.7%)
Verbal communication	0 (0%)	117.3 (48.5%)
Touch	0 (0%)	70.6 (29.2%)
Aggressive behavior	13.6 (66.3%)	6.3 (0.3%)
Acceptance of care	No	Yes

**Table 4:** Ratio of communication elements during care.

Case	Conventional care				Humanitude care			
	1	2	3	Average	1	2	3	Average
Total care time (sec)	300.5	360.7	20.5	227.2	320.2	127.6	241.8	228.1
Eye contact (%)	1.7	0	0	0.6	19.5	4.3	13.7	12.5
Verbal communication (%)	19.7	27.4	0	15.7	73.9	42.1	48.5	54.8
Touch (%)	0.2	0	0	0.1	60.4	44.0	29.2	44.5
Aggressive behavior (%)	25.0	24.5	66.3	38.6	0	0	0.3	0.1
Acceptance of care	No	No	No		Yes	Yes	Yes	

**Table 5:** Subanalysis: number of modalities used simultaneously in care.

Number of modalities applied simultaneously	Conventional care %			Humanitude %		
	Case 1	Case 2	Case 3	Case 1	Case 2	Case 3
0	76.3	69.2	100	13.8	21.5	13.6
1	23.1	29.7	0	35.2	42.3	32.2
2	0.5	1.1	0	32.7	35.4	20.1
3	0.1	0	0	19.7	0.8	3.7
